# Risk of violence from a current or former partner: Associated factors and classification in a nationwide study in Colombia

**DOI:** 10.1371/journal.pone.0279444

**Published:** 2022-12-22

**Authors:** Isaac Esteban Camargo Freile, Karen Cecilia Flórez Lozano, Carlos Alberto Sarmiento Crespo, Carolina Mercedes Vecchio Camargo, Sandra Milena Rodríguez Acosta, Victor Florez-Garcia, Edgar Navarro Lechuga

**Affiliations:** 1 Department of Mathematics and Statistics, Universidad del Norte, Barranquilla, Colombia; 2 School of Basic Sciences, Technology, and Engineering, Universidad Nacional Abierta y a Distancia–UNAD, Barranquilla, Colombia; 3 National Institute of Legal Medicine and Forensic Sciences—North Regional, Bogota, Colombia; 4 Department of Economics, Universidad del Norte, Barranquilla, Colombia; 5 Department of Public Health, Universidad del Norte, Barranquilla, Colombia; 6 Joseph J. Zilber School of Public Health, University of Wisconsin -Milwaukee, Milwaukee, WI, United States of America; Public Library of Science, UNITED STATES

## Abstract

Intimate partner violence (IPV) includes assaults that risk a woman’s bodily integrity. Intimate partners commit IPV, people with whom the victim shares (or shared) a close personal or sexual relationship. This phenomenon has a great global and national impact. Thus, it is necessary to establish trends of the risk of physical violence to women by their current or former partner in each department of Colombia and its relationship with sociodemographic and health characteristics. This study uses an ecological approach at the departmental level, with victims of intimate partner violence treated at the National Institute of Legal Medicine and Forensic Sciences (INMLyCF). Potential factors were identified through Bayesian factor analysis and were included in the model to estimate risk. The findings show that the Casanare department had the highest risk of producing victims (SMR: 2.545). In departments where the educational level of women is at or below primary school, there is a high-risk β = 0.343 (0.285, 0.397) of them being assaulted. For the departments in which the employment of women is in sales and services or office workers, the associated factor presents a higher risk β = 0.361 (0.201, 0.485), as in the risk related to affiliation with the social security system β = 0.338 (0.246, 0.498), as well as sexual and reproductive life β = 0.143 (0.003, 0.322). The following categories were associated with physical gender violence: no education and low participation in making purchases at home β = 0.106 (0.049, 0.199), low participation in decisions about their health, and visits to family and friends β = 0.240 (0.170, 0.299). Therefore, public health programs should strengthen women’s empowerment in household decisions and increase their educational level to reduce this incidence.

## Introduction

In the Declaration on the Elimination of Violence against Women, the United Nations [1] defined violence against women as “any act of violence based on gender, that results or may result in physical, sexual, or psychological harm or suffering for women, as well as threats of such acts, coercion, or arbitrary deprivation of liberty (…).” These types of violent acts are verified by other authors [2]. Therefore, intimate partner violence (IPV), which can take any of the forms above, is violence committed by current or former intimate partners [3], that is, people with whom a close personal or sexual relationship is (or was) sustained, whether or not they are a same-sex couple [4]. However, in most cases of IPV, the perpetrator is male [5,6].

Worldwide, over 800 million women have experienced physical and sexual violence by an intimate partner [7]. In 2017, women represented 82% of homicide victims exclusively perpetrated by an intimate partner [6]. However, evidence indicates a higher prevalence of IPV in low- and middle-income countries. For example, approximately 30% of women in Latin America and the Caribbean have reported having been exposed, throughout their lives, to physical and sexual violence. Additionally, in high-income countries such as Australia, Canada, and the United States, the percentage is 23% [8].

Colombia shares a level of prevalence of intimate partner violence against women similar to that of Mexico, Costa Rica, Nicaragua, El Salvador, and Honduras, of approximately 30%. This is surpassed by Ecuador, Peru, and Bolivia, countries that present the highest prevalence in the region, with 40% [8]. It is worth mentioning that between 2007 and 2017, women were the primary victims of IPV, with approximately 80% of the cases registered in Colombia. In 2009, the highest rate of 135.91 per 100,000 inhabitants was recorded, while 2013 had the lowest rate of 95 per 100,000 inhabitants. The period between 2013 and 2017 showed a marked geographical differentiation regarding IPV, with Antioquia, Bogotá, Cundinamarca, and Valle del Cauca constantly registering the highest number of cases [9,10].

From a regulatory perspective, with the introduction of Law 1257 of 2008 [5], a specific regulation was introduced for the first time in the country for the “awareness, prevention, and punishment of forms of violence and discrimination against women.” Later, partial regulations were issued based on this law, such as Decree 4463 of 2011 [11], Section 1; Decree 4796 of 2011 [12], Section 1; Decree 4798 of 2011 [13], Section 1; and Decree 4799 of 2011[14], Section 1 [14]. These caused increased campaigns to recognize women’s rights and awareness-raising and the prevention of gender-based violence. [15].

In the presence of any case of physical IPV, the National Institute of Legal Medicine and Forensic Sciences (INMLyCF), by written request of the competent authority or by articles 267 and 268 of Law 906 of 2004 [16], is tasked with appraisal via medical-legal examinations.

Considering the spatial dynamics of physical violence against women, the empirical evidence reveals that at the levels relative to the community their socioeconomic situation is one of the factors that most influences the prevalence of this phenomenon. IPV occurs in all social groups, but it predominates in disadvantaged homes and neighborhoods under precarious conditions, characterized by low service infrastructure and low collective effectiveness (social organization and capacity to control problems), unemployment, and poverty [17,18]. Thus, through the socioecological perspective, the study of the risk of being a victim of physical violence requires determining the level of aggregation of the different social areas under analysis, deepening the study of socioeconomic factors that are relevant and transversal and that can result in variations of risk rates [19–21].

Legal and social sciences define four dimensions of crime: 1. Legal, a law is broken, 2. Victim, someone or something is the object of the crime, 3. Offender, someone commits the crime, and 4. Spatial, the crime happens somewhere. This last dimension not only refers to the fact that crime has an inherent geographical quality, but it also means that it can be better understood when the components associated with this quality are explored.

In the last decade, epidemiological studies have highlighted the importance of spatial distribution regarding the social approach of scientific research. In this field, statistics play a remarkable role, and the Bayesian approach represents an advance in studying spatial relationships that underlie these phenomena.

Social characteristics are highly relevant in some studies on the correlations between neighborhood characteristics and the occurrence of IPV [22,23]. Scholars have found that some aspects of the neighborhood, such as poverty, ethnicity, and others, can better approximate the behavior of criminal violence than that of IPV, which they conclude is more determined by personal characteristics.

Gracia et al. [24] analyzed a dataset related to IPV and child abuse in Valencia, Spain. They estimated the risks of each type of violence and included characteristics of social contact with population statistics and observational characteristics.

A study of women of reproductive age in Rwanda showed that the factors that determine the risk of domestic violence against them are the wealth quintile of the household, the size of the home, the age of the partner and their educational level, polygamy, alcohol consumption, use of contraceptives, and others [25].

These studies have shown that the spatial distribution of violent crimes is not random but rather that areas with higher crime rates exist. These areas are associated with sociodemographic characteristics that allow for characterizing the crime. For this reason, the analysis of patterns through the Bayesian approach is particularly suitable for analyzing small areas since it will enable incorporating geographic information and making maps of the spatial components that express the risk variables according to the site.

This work establishes the patterns/trend of the risk of physical violence against women by their former or current partner in departments in Colombia during the 2013–2017 period and examines its relationship with sociodemographic characteristics, household decisions, educational level of women, economic activity, affiliation with social security, employment, migration, use of contraceptive methods, and others.

## Methodology

### Study population

This study was based on ecological analysis, where the analysis units were the 32 administrative departments of Colombia and the Bogotá Capital District (DC). These records included victims assessed for violence by a former or current partner after a court order who went to the INMLyCF facilities in the 32 departments of Colombia and Bogotá DC from 2013 to 2017. Only the cases where the victims were women were considered. Additionally, we included datasets from Forensis Data for Life (reported cases of physical violence against women) [9,10,26–28]; National Survey of Demography and Health 2015 [29]; National Administrative Department of Statistics [30], and National Planning Department [31]. The variables in this study were extracted from previous records from the National Survey of Demography and Health 2015 Volume I and II [29]; National Administrative Department of Statistics [30], and National Planning Department [31].

### Measures

The dependent variable in this analysis was physical violence against women (Yes vs. No). These women are aged 13 to 49 years old. Additionally, we included household decisions: the proportion of women who alone or along with someone else have the last word in specific household decisions among seven categories: personal health, large household purchases, daily household purchases, visiting relatives or friends, everyday meals, all of the above, none of the above. Their educational level was measured as no education, incomplete primary school, complete primary school, incomplete secondary school, complete secondary school, and higher education. Employment type was measured as professional/technician/manager, office worker, sales and services, qualified manual labor, unskilled manual labor, and agriculture. Affiliation to social security was measured as a contributory health scheme, subsidized health scheme, exception, special, not affiliated, or do not know. Employment was estimated as currently employed, without a current job, or not employed for the last 12 months.

Migration. Relationship with a migrant person, which was measured as the proportion of international emigrants with a relationship with the head of the household, included the following categories: partner, child, mother/father. The reason for migration was measured as being a sibling, education, work, exile, marriage, other, do not know, the number of migrants.

Sexual education. Additionally, the use of contraceptive methods was measured in total modern contraceptive methods, some traditional contraceptive methods, or not currently used. Voluntary use of contraceptive methods was measured as having been forced to use a contraceptive method and knowing that health providers offered free temporary methods. The number of children was categorized as "average children per birth-giving woman, 40–49 years" and "average children per woman, global fertility rate 15–49 years." Age of first sexual intercourse was measured as the proportion of women who had sexual intercourse before age 15: "the percentage who had sexual intercourse before age 18." The information level on promotion and prevention was measured as the proportion of women who reported data in the following categories: Pap smear, breast self-examination, and sexually transmitted infections such as HIV. Condom use requirement was measured as the proportion of women aged 13–24 who used a condom during the last sexual intercourse. Recognition of the rights of same-sex couples was measured as the proportion of women 13–49 years old and men 13–49 years old who believe that homosexual people have the same rights as heterosexual people. Adoption by same-sex couples was measured as the proportion of women 13–49 years old and men 13–49 years old who agreed with homosexual couples adopting children.

We explored the acceptance of violence according to the following categories: women who are currently in a relationship and reported their partner, sometimes it is right for men to hit their partners, it is justified to hit a partner when she has been unfaithful to them, and women who continue with their partners after being hit. Additionally, the responsibility for household economic decisions was categorized as only the woman making decisions, both, someone else intervening, and only the partner. The participation of women in household expenses was measured as nothing, almost nothing, less than half, half, more than half, everything.

Additionally, we set up an index on barriers to access to the labor market due to gender issues among women who have ever worked, the proportion of women who have required a pregnancy test, sterilization certificate, or AIDS test, and the proportion fired while pregnant. Additionally, pregnancy test requirements for a job position, sterilization certificate requirements for a job position, HIV test requirements for a job position, and dismissal for pregnancy were recorded.

We included economic indicators such as the proportion of people in extreme monetary poverty, the monetary poverty gap, the share of gross domestic product by the department at current prices, the share of gross domestic product by the department, the share of gross domestic product by the department at constant prices in 2015, and the annual percentage change at regular 2005 prices.

### Statistical analysis

Descriptive statistics were used to characterize the distribution of the study variables for the total reported cases of physical violence against women in Colombia. Charts and descriptive metrics are used, including percentages, measures of central tendency, and dispersion. Then, a simple correlation analysis between the variables with higher prevalence categories was conducted to identify potential grouping factors through Bayesian factor analysis. We applied factor analysis using exploratory Bayesian methodology due to a large number of variables and to reduce dimensions [32]. This method simultaneously selects the dimension of the factorial model, the assignment of the measurements to the factors, and the factorial loading [33]. The number of elements is not determined as a first step but is inferred along with other parameters. Factorial identification was carried out through the Metropolis‒Hastings algorithm using R software [34].

The measurement of the risk of gender violence was performed through the standardized morbidity ratio (SMR), which relates the value ​​of observed cases (number of women injured by a current and former partner, appraised by INMLyCF, 2013–2017) and the number of expected cases in a particular area if all areas had the same risk as the general population. However, these rates are unstable and sometimes show significant variability, as they depend on the population size of each area under study. In addition, its representation on a map exhibits a wide and uninformative variation of existing patterns [24].

Thus, spatial models included in disease mapping theory are commonly used, which allow for studying the different spatial relationships of risk. The Besag, York & Mollié (BYM) convolution model was implemented to study these spatial relationships [35,36]. The model assumes that neighboring areas have common risk factors and, therefore, a spatial relationship exists between them.

The average risk in each area was explained by the risk in its neighboring regions. The information provided by each area that shares at least one geographic point with the site for which the risk is estimated provides elements that explain the risk behavior in an area under study.

At the first level of the hierarchy, the model assumes that the observed counts of violence in the i-th area *y_i_* follow a Poisson distribution:

$$y_{i}/e_{i}\theta_{i}∼Po(e_{i}\theta_{i}),con\;i=1,\ldots ,m$$ (1)

where *e_i_* is the number of cases expected in the i-th area, and *θ_i_* is the relative risk in the i-th area.

The parameter of interest is the relative risk *θ_i_*, so that the second level of the hierarchy is defined as:

$$log(\theta_{i})=\alpha_{0}+\Sigma {{X\prime }_{i}\beta }_{i}{+u}_{i}{+v}_{i}$$ (2)

This equation allows for modeling from correlated and uncorrelated random effects, where *X*′_*i*_*β_i_* is a linear predictor, *α*_0_ contains the common risk in the study area, *u_i_* is the random component with spatial dependence, and *v_i_* is the term that corresponds to uncorrelated heterogeneity.

An uninformative uniform distribution is used for the linear predictor *β* and the *v_i_* and *α*_0_ components, while for the spatially structured term *u_i_*, a conditional spatial autoregressive model (CAR) is used.

Markov Chain Monte Carlo (MCMC) simulation techniques are applied for Bayesian inference. These analyses were performed using WinBUGS and RStudio software [34,37]. A total of 10,000 iterations were generated in each model, and the first 4,000 were considered a warm-up. Additionally, the convergence was graphically evaluated for all parameters.

### Ethics statement

This study met the ethical considerations of human research. The Institutional Review Board (IRB) by the Universidad del Norte approved the protocol according to minute 180 (2018). Additionally, the authors anonymized the complete dataset to hide the confidential information of every subject.

## Results

**Fig 1:**
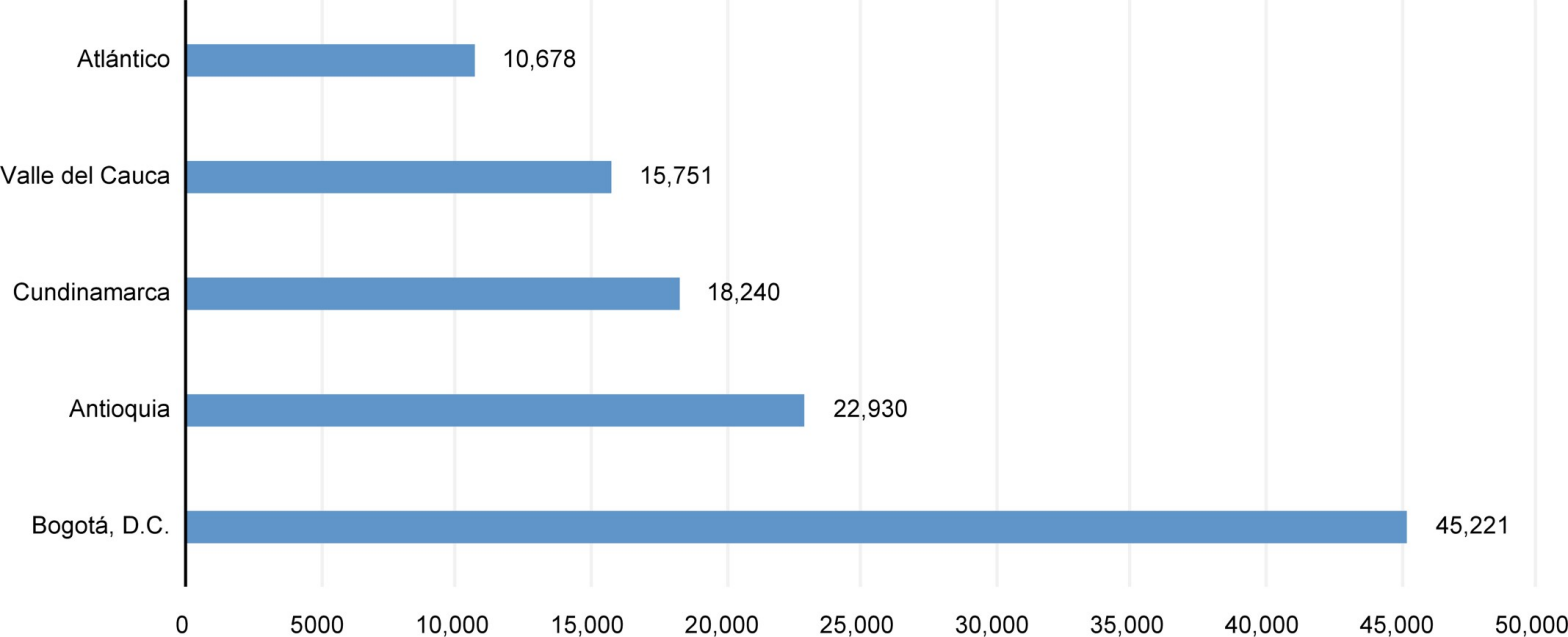
Distribution of cases of physical violence against women in territorial entities with the highest frequency during the period of the study. Sources: Forensis (2013–2017).

The number of women injured by a current or former partner attended by the INMLyCF from 2013 to 2017 was 208,700. Fig 1 depicts the distribution of the five territorial entities, four departments, and the capital district, with the highest number of cases in this period.

Most women (82%) were between 20 and 44 years old, and most (40%) had attended elementary or high school compared with 2.9% who had a university degree and 13.6% with a technical degree. The predominant marital status was free union at 45.5%. The most frequent alleged aggressor was the permanent partner at 46%.

Regarding the distribution of the covariates measured at the population level, on average 97.8% ± 5.1% have information on vaginal cytology, 78.2% ± 5.3% of women make decisions about their health, and 73.1% ± 5.3% make decisions regarding visits to family and friends. The mean percentage of women who knew that health providers offered free temporary methods was 58.3% ± 9.5%. (Table 1).

**Table 1:** Descriptive analysis of the variables with of the categories with the highest proportion.

Variable	Median (%)	SD (%)	Min	Max
**Household decisions**				
Personal health care	78.2	5.3	64.7	87.1
Big purchases	58.6	5.2	36.4	68.1
Daily purchases	61.2	4.3	47.1	69.5
Visiting family and friends	73.1	5.3	55.5	81
Everyday meals	67.7	2.9	59.6	73.9
**Economic activity**				
Sales and services	60.3	10.4	22	81.5
**Use of contraceptive methods**				
Total, modern contraceptive methods	72.9	8.9	37.8	81.5
**Voluntariness on the use of contraceptive methods**				
Knowing that health care providers offer free contraceptive methods	58.3	9.5	26.8	76
**Age of first sexual intercourse**				
Proportion of women that had sex when younger than 18	68.7	9.7	53.8	84
**Women with pap-smear information**				
Proportion of women that know	97.8	5.1	72.7	97.9
**Agrees with men hitting them in specific situations**				
Women that stay after their partner hits them do it because they like it	60.2	6.7	40.9	70.8
**Responsible of household decisions**				
Only the woman	62.2	6.9	45.3	75.5

SD, standard deviation.

**Fig 2:**
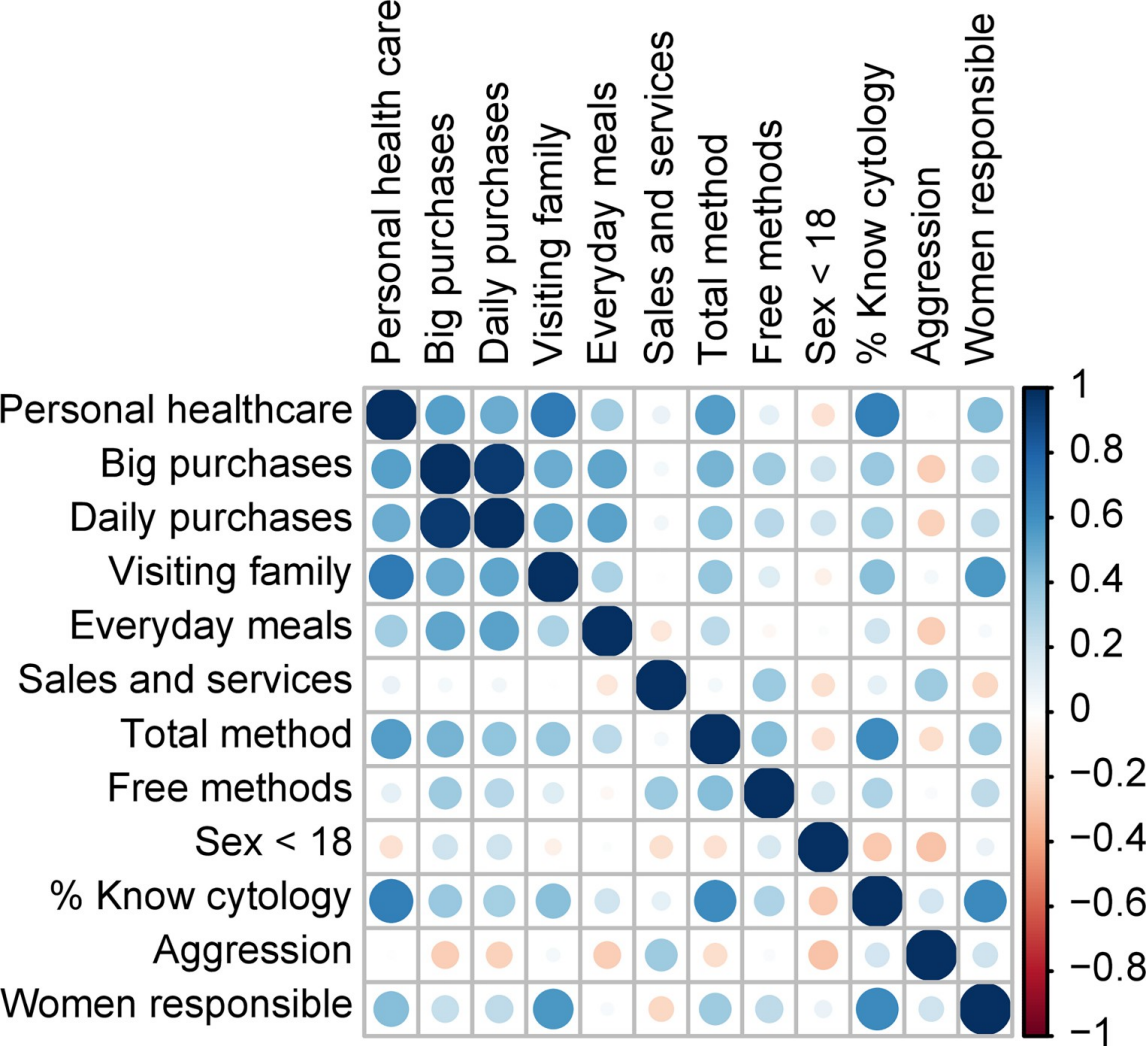
Correlation for variables with higher prevalence categories. **Visiting family:** Visiting family and friends. **Total method:** Total modern contraceptive methods include female sterilization, male sterilization, contraceptive pills, intrauterine devices (IUDs), monthly contraceptive injections, quarterly contraceptive injections, contraceptive implants, male condoms, contraceptive foams/jellies/sponges, and the lactational amenorrhea method (LAM). **Free methods:** Knowing that health care providers offer free contraceptive methods**. Sex<18:** Proportion of women who had sex younger than 18. **% Know cytology**: Proportion of women who have pap smear information. **Aggression:** Agrees with men hitting them in specific situations; women who stay after their partner hits them do it because they like it. **Women are responsible:** Households where women are responsible for decisions.

Fig 2 depicts the correlation analysis between the study variables with the highest proportion. The greater the intensity of the color and the size of the circle, the greater the association. For example, the categories use of contraceptive methods and large household purchases showed a high positive correlation (ρ = 0.77). This finding suggests that women who make decisions on household expenses potentially purchase and use contraceptive methods.

There was a high positive correlation between the percentage of women managing their health and making large household purchases (ρ = 0.75). This suggests that women in charge of household expenses oversee decisions regarding their health or are self-sufficient in this area.

There was a high positive correlation between women who knew about cytology and total modern contraceptives (ρ = 0.87). This may indicate that women who use modern contraceptive methods identify the importance of vaginal cytology to detect diseases on time.

Due to many sociodemographic, sexual, and reproductive life and macroeconomic variables, a BEFA Bayesian factor analysis was conducted to reduce their number and create factors that group correlated variables [33]. Thus, seven factors presented significant weight for their inclusion in the model and were defined as follows:

For the factor defined as general characteristics of the household, two factors were generated as g*eneral characteristics of the household I*, made up of the categories own health, visits with family and friends, do not make decisions and *general characteristics of the household II*, made up of the categories without education, large or daily household purchases, makes all the decisions. Additionally, the other factors were set up based on different factorial charges (Table 2).

**Table 2:** Description of factors identified in the Bayesian Exploratory Factor Analysis (BEFA).

		Factorial charge	
Factor	Covariable	Negative (-1)	Positive (1)
1	General household characteristics I	Do not make decisions	Personal health care, visiting Friends and family
2	General household characteristics II	No education	Big or daily purchases, makes decisions
3	Educational level	Finished high school, further education	Primary school (finished or unfinished)
4	Economic activity	Agriculture	Sales and services, office work
5	Social security	Subsidized coverage, special health scheme	Contributory health scheme
6	Reproductive and sexual life	Does not use contraceptive methods, average children 15–49 years and 40–49	Uses modern contraceptive methods, knows that health care providers offer free contraceptives
7	Sexual life, fecundity, and knowledge index	Information on pap smear, information on breast self-exam, comprehensive knowledge of HIV	Average children 15–49 years and 40–49, first sexual intercourse before the age of 15, does not know of STIs

The weight of the rest of the variables in the factorial analysis was considered negligible (Metropolis‒Hastings acceptance index of less than 1) [33], implying the absence of a factorial structure. Therefore, they were included in the BYM model separately without factoring to identify the best submodel. The inclusion of the percentage of investment in education stands out, which allowed for a greater number of statistically significant factors in the BYM model.

**Fig 3:**
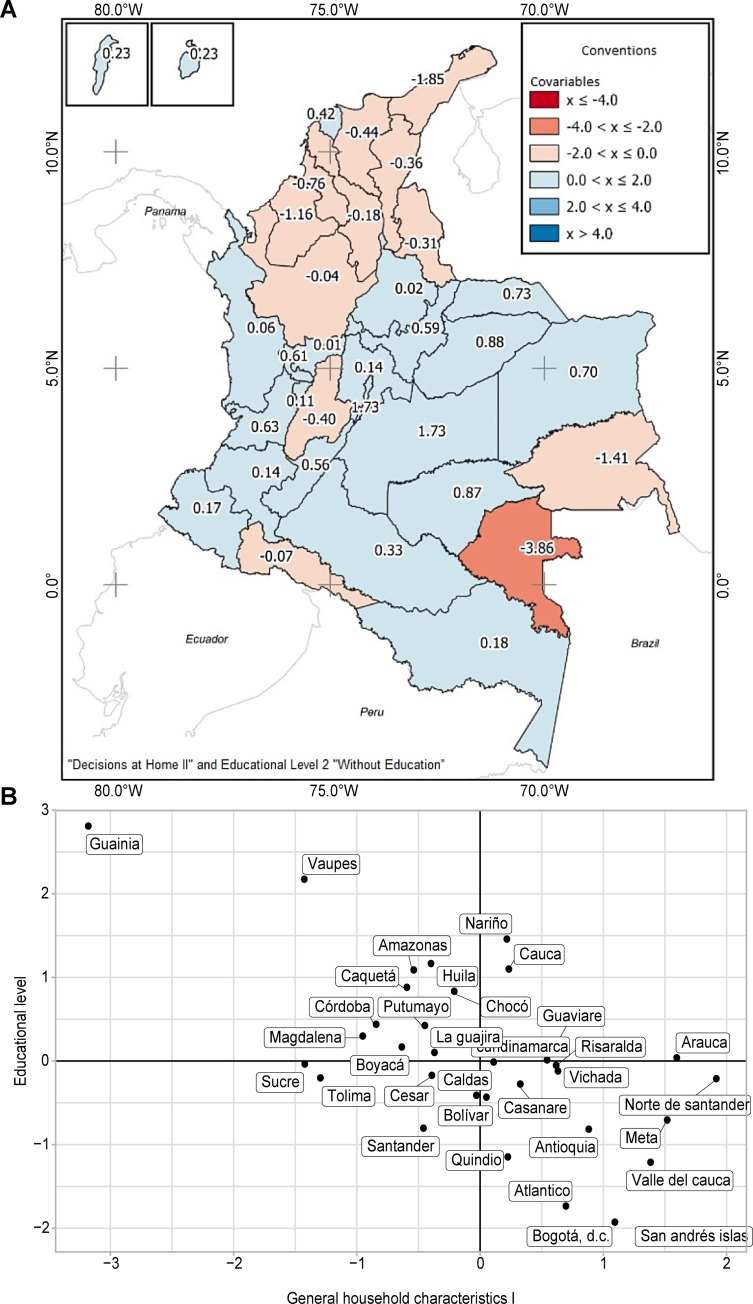
**a. Spatial distribution of BEFA analysis "Decisions at Home II" and Educational Level 2 "Without Education”.** Reprinted from [38] under a CC BY license, with permission from [Agustin Codazzi Geographical Institute—IGAC], original copyright [2020]. **b.** Biplot diagram between identified factors spatial distribution BEFA analysis "Household Decisions I" and educational level.

The BEFA analysis allowed for the classification of the departments, keeping related factors such as Factor 2 associated with the *general characteristics of household II*. The departments with negative scores in that factor were those with a higher proportion of "no education." The positive scores of the factor correspond to women making household decisions about purchases or in general. For example, departments with redder tones, such as Vaupés, indicate a high negative score (further from zero). Thus, the proportion of women without education in that department was high, and the proportion of women who made purchase decisions was very low. That is unlike areas with blue tones, such as Bogotá or Meta, where the proportion of women without education was low. The proportion of women who made decisions at home was high (Fig 3A).

Fig 3B illustrates the typification of the areas considering the relationship between the household decisions I factor and educational level, where the closest departments present a relationship if and between the categories of each factor. The further from zero, the higher the proportion of women in that category of each corresponding factor (Fig 3B) [38].

For the lower right quadrant, there was a group of areas where women had an education level, completed secondary school, or had higher education. Additionally, they make decisions at home about their health and visit family and friends. Similarly, in areas such as Bogotá or Atlántico, the proportion of women without education was low, and the proportion of women who made decisions at home was high.

In the upper right quadrant, there were areas where women had an incomplete or complete primary education level and, in turn, made decisions at home about their health and visiting family and friends. As in Nariño and Cauca, the proportion of women with incomplete primary education was low, and the proportion of women who made decisions at home was high.

In the upper left quadrant are the areas where women have an educational level, complete or incomplete primary school, and do not make decisions at home, such as the departments of Vaupés and Guainía, where the proportion of women with incomplete primary education is high.

Finally, we observe those areas where the completed educational level was secondary or higher in the lower-left quadrant. However, they have no participation in decision-making at home. Additionally, in Santander, the proportion of women with a secondary education level was low and did not participate in decision-making at home.

**Fig 4:**
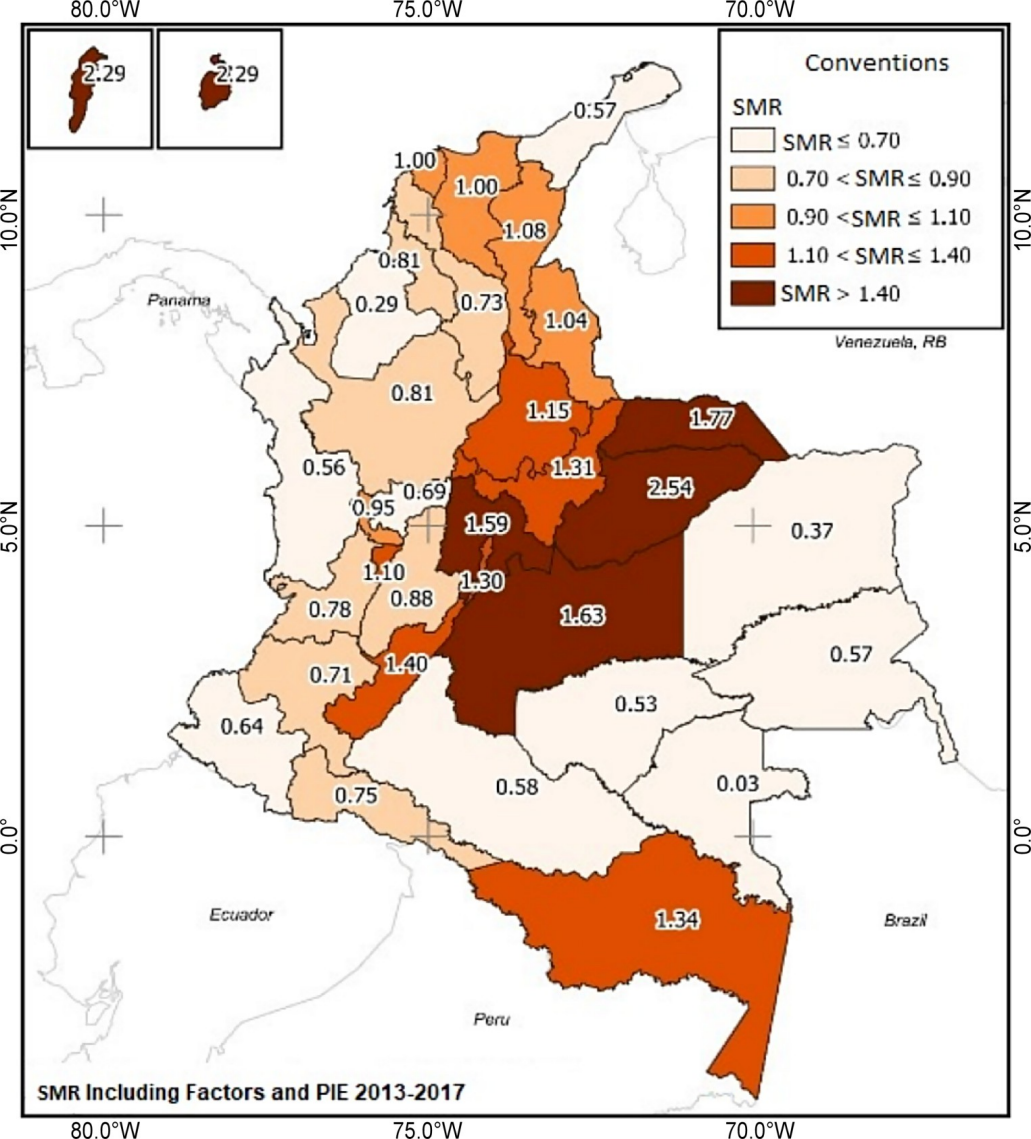
Spatial distribution of the risk of physical violence against women Colombia, 2013–2017, including factors and percentage of investment in education. **PIE**: Percentage of investment in education. Reprinted from [38] under a CC BY license, with permission from [Agustin Codazzi Geographical Institute—IGAC], original copyright [2020].

After implementing the Bayesian factor analysis, the population risk of being injured by a former or current partner was modeled in each Colombian department using disease mapping methods from a Bayesian approach to estimate risk. For each area, the average value of the risk was obtained. The map exhibits a pattern with medium- and high-risk values from the center to the country’s south. Values ​​above one convey a greater risk. Therefore, the highest risk of injuries in women victims of physical violence was found in the departments of Casanare (SMR: 2.545), San Andrés, Providencia y Santa Catalina (SMR: 2.3), and Arauca (SMR: 1.773) (Fig 4) [38].

Table 3 shows the mean, the standard deviation, and the credibility interval observed for each of the factor coefficients of the model. The mean of the coefficient (beta) with a positive value indicates the presence of a factor that increases the risk of physical violence against women. For the *general household characteristics I* and *general household characteristics II*, the value is positive. It has a statistically significant association β = 0.106 (0.049, 0.199) and β = 0.240 (0.170, 0.299), respectively, which means that in the departments where women have little or zero decision-making power regarding their health, visiting family and friends, and large or daily household purchases, they are at greater risk of physical violence.

**Table 3:** Results of the BYM Bayesian model for physical violence against women, 2013–2017, including factors and percentage of investment in education.

Covariables	β (Mean)	SD	β (95% lC)
Intercept	-0.176	0.053	-0.296, -0.092
General household characteristics I	0.106	0.034	0.049, 0.199
General household characteristics II	0.240	0.033	0.170, 0.299
Educational level	0.343	0.032	0.285, 0.397
Economic activity	0.361	0.076	0.201, 0.485
Social security	0.338	0.075	0.246, 0.498
Reproductive and sexual life	0.143	0.094	0.003, 0.322
Sexual life, fecundity, and knowledge index	-0.072	0.098	-0.227, 0.058
Percentage of investment—Education	0.004	0.002	0.001, 0.009

SD, standard deviation, lC, interval of credibility.

In departments where the educational level of women is an incomplete or complete primary school, there is a significantly high risk of being a victim of physical violence β = 0.343 (0.285, 0.397). For departments where the women´s employment type was sales and services, or office workers, the associated factor presents a significantly high risk of physical violence β = 0.361 (0.201, 0.485). In the case of affiliation with social security, subsidized health schemes, special health schemes, and contributory health schemes, the associated factor presents a significantly higher risk of physical violence β = 0.338 (0.246, 0.498).

The factor associated with *affiliation with social security*, *sexual and reproductive life* presents a significantly higher risk of physical violence against women β = 0.143 (0.003, 0.322). Furthermore, the percentage of investment in education was statistically significant β = 0.004 (0.001, 0.009). This implies a greater risk of physical violence in those departments where the investment destined for education is lower. Additionally, no effect was observed on the model for the factor associated with sexual life, fertility, and knowledge index β = -0.072 (-0.227, 0.058).

## Discussion

In this study, we utilized an approach based on spatial epidemiology to analyze the relationship of characteristics such as the educational level of women, economic activity, affiliation with social security, and reproductive life. The results have shown that the risk of intimate partner violence against women follows a spatial pattern. Additionally, there is evidence that the risk for this IPV is higher or lower in departments depending on women’s occupations.

The results show that Colombia’s departments with the highest risk values ​​are Casanare, San Andrés, Providencia y Santa Catalina, and Arauca, with SMRs between 1.77 and 2.54. Low educational levels and lack of decision-making at home increased the risk of physical violence against women in these departments.

Gracia et al. [24] found that IPV is found in people who have a low educational level and live in low socioeconomic strata. In these studies, the relationship is observed at the **individual** level, while in the present study, this situation is transferred to the aggregate level in the different departments.

This study shows a high correlation between categories such as the decision power to make purchases at home and the use of modern contraceptive methods, which negatively impact the risk of violence against women by a current or former partner at the departmental level.

From these maps, we identified areas with a greater risk of IPV. Furthermore, this study supports previous research about the geographical location where the event occurs and is important to explain the risk pattern of being attacked by a current or former partner [22,24]. Likewise, it provides evidence that the chances for this type of violence are simultaneously high or low in departments with characteristics such as a low educational level of women, economic activity (sales and services), affiliation with social security and reproductive life.

Those departments where women do not make decisions, or they have little participation in aspects associated with their rights and basic needs, have a greater risk of violence against women by their current or former partners. Although women are exposed to IPV, studies show that some women are more exposed to violence than others [39–41]. The primary factors that determine the social status of women are education, employment, and social security. In studies conducted in other countries, women’s participation in education and business life is hampered by social contexts at the family level [42]. One of the most critical risk factors for violence against women is a lower educational level [39,41,43]. As the academic level of women decreases, the rates of exposure to violence increase. While one in five women with higher education is exposed to violence, one in two women with a lower education degree is exposed to violence. [42] In our study, the departments with the highest proportion of women with low educational levels correlate with increased rates of violence by a current or former partner.

We did not observe any effect in the model regarding the characteristics associated with sexual life, fertility, and the knowledge index. Finally, this study has strengths and limitations. First, we performed the spatial analysis of the risk of being attacked by a current or former partner, alongside covariates of different natures and levels. Using appropriate Bayesian statistical techniques from disease mapping provides a better understanding of the spatial pattern of the studied risk. Regarding our limitations, the underreporting of cases of IPV is because not all of them are reported to and therefore treated by INMLyCF. The data were obtained at the departmental level. Thus, it would be appropriate to have a more detailed level of aggregation (municipality, neighborhood, block) that allows for a greater approximation of the characteristics and context related to the event. Regarding the limitations of the covariates, other socioeconomic measures are absent, such as average monthly family income, the proportion of families with their own home, percentage of financial and commercial activities, and other variables of importance, such as physical disorganization, the concentration of immigrants, police activity, alcohol outlets, among others.

## Conclusion

The estimated risk of violence against women had a heterogeneous variation throughout the national territory, with a higher concentration in the departments of Casanare, San Andrés, Providencia y Santa Catalina, and Arauca. The factors associated with a higher risk are educational level and low participation in decision-making about their health, visiting family and friends, and large or daily household purchases, which are determinants of this type of violence. Therefore, public health programs must design interventions focused on strengthening women’s empowerment in household decisions and increasing their educational level as critical points to reduce the incidence of violence.

The results constitute an advance in the study of violence against women that will later allow for the inclusion of the effect of context covariates such as the consumption of psychoactive substances, alcohol, and family income.
